# ROC Analysis to Improve Sensitivity and Specificity of the PlusoptiX Photoscreener

**Published:** 2012-01

**Authors:** David Silbert, Noelle Matta

**Affiliations:** Family Eye Group, Lancaster, PA, USA

The current issue of JOVR includes an article entitled “Is Noncycloplegic Photorefraction Applicable for Screening Refractive Amblyopia risk Factors?”.^1^ This is a well-constructed study comparing noncycloplegic photorefraction utilizing the plusoptiX SO4 screener to cycloplegic autorefraction (or cycloplegic retinoscopy in those few children in whom cycloplegic autorefraction could not be performed) in detecting AAPOS defined amblyopia risk factors. The authors compared refractive readings of the right eye from 185 children.

The authors found an agreement rate of 89.7% between noncycloplegic photoscreening and cycloplegic autorefraction/retinoscopy in determining AAPOS defined amblyopia risk factors. They then broke out myopia, hyperopia, and cylindrical power and analyzed them separately. While the authors found that sensitivity and specificity were quite good for both myopia and astigmatism, sensitivity was poor for hyperopia. This is not surprising given the accommodative state present in noncycloplegic photoscreening.

Through ROC analysis, the authors were able to propose new cut-offs for the plusoptiX photoscreener. By changing the cutoff for hyperopia to +1.87D they were able to significantly improve the sensitivity of the test while only minimally impacting specificity for determining AAPOS defined amblyopia risk factors. They were also able to make a slight change in the cutoffs for astigmatism; decreasing the cutoff from +1.50 to +1.12D improved sensitivity while minimally affecting specificity. Using the authors’ new cut-off values they were able to improve sensitivity and specificity from 79.0% and 94.5% respectively, to 91.2% and 82%.

There are some limitations to the study. While the authors note that cycloplegic refraction is the gold standard for detecting refractive errors in children, they actually utilized cycloplegic autorefraction in place of cycloplegic refraction in 167 (90%) of children, and performed cycloplegic retinoscopy in only the 18 children whom autorefraction could not be performed. Choong et al showed fairly good but no absolute correlation between cycloplegic autorefraction and cycloplegic manifest refraction, noting excellent correlation in myopia, but slightly poorer correlation for hyperopia.^2^ It is unlikely however, that the use of cycloplegic autorefraction (instead of cycloplegic refraction as the gold standard) would materially alter the results of this study.

In this study, the authors have provided a useful mechanism for clinicians to adjust cut-offs for the plusoptiX photoscreener, customized to the needs of their specific screening programs. By adjusting individual cut-offs for hyperopia, myopia and cylinder, programs can fine-tune their results altering sensitivity, specificity and referral rates.
